# Coronavirus neuropathogenesis: could SARS be the tip of the iceberg?

**DOI:** 10.1186/1753-6561-2-s1-s40

**Published:** 2008-09-23

**Authors:** Pierre J Talbot, Marc Desforges, Julien St-Jean, Hélène Jacomy

**Affiliations:** Laboratory of Neuroimmunovirology, INRS-Institut Armand Frappier, Laval, Québec Canada H7V 1B7

**Keywords:** Encephalitis, Severe Acute Respiratory Syndrome, Common Cold, Neurologic Disease, Severe Acute Respiratory Syndrome

Ubiquitous worldwide, coronaviruses are known to cause serious and economically damaging respiratory, enteric and neurologic diseases in animals such as cattle, pigs and fowl. Since the mid-1960s, they are recognized human respiratory pathogens involved in up to a third of infections of the upper respiratory tract, of the common cold type. Even though they are suspected to cause more serious infections in humans, affecting the lower respiratory and gastro-intestinal tracts, as well as the central nervous system (CNS), this remained subject of investigations in only a few laboratories. This changed dramatically in 2002–2003, when the Severe Acute Respiratory Syndrome (SARS) epidemic swept the world, and was linked to a coronavirus (SARS-CoV) that evolved in bats, before being transmitted to humans in Southeast Asia, with some reports of neurological involvement.

We have accumulated evidence that human coronaviruses (HCoV) may cause neurologic disease after CNS neuroinvasion and persistence. Strain OC43 readily established persistent infections of neural cell lines and acquired mutations in the gene encoding the viral surface protein S, which interacts with cellular receptors. Biologically cloned emerging virus variants had mutations in the putative S receptor-binding domain, which correlated with the ability to infect not only neurons, the main target cell type, but also astrocytes. Four reproducibly observed S mutations were introduced into an infectious cDNA clone (rOC/ATCC) to yield rOC/U and the two recombinant viruses were compared for the induced neuropathogenesis in BALB/c mice. We found that susceptibility to rOC/U infection was enhanced and mice showed hind limb paralysis associated with spinal cord demyelination, whereas rOC/ATCC induced encephalitis.

These results suggest that acquisition of discrete S mutations after persistence of HCoV-OC43 in neural cells results in modulation of neurovirulence and modified neuropathology. This may have implications in our understanding of coronavirus pathogenesis in humans, and points to the need for global research, prevention and intervention efforts targeting various infectious pathogens that could unexpectedly cause a global threat.

Funded by the Canadian Institutes of Health Research, Canada Research Chair.

